# Secondary Distribution of HIV Self-Testing Kits to Social and Sexual Networks of PLWH in KwaZulu-Natal, South Africa. A Brief Report

**DOI:** 10.3389/fpubh.2022.855625

**Published:** 2022-04-27

**Authors:** Nsika Sithole, Olivier Koole, Kombi Sausi, Meighan Krows, Torin Schaafsma, Alastair Van Heerden, Maryam Shahmanesh, Heidi van Rooyen, Connie Celum, Ruanne V. Barnabas, Adrienne E. Shapiro

**Affiliations:** ^1^Clinical Research Department, Africa Health Research Institute, Somkhele, South Africa; ^2^London School of Hygiene and Tropical Medicine, London, United Kingdom; ^3^Human Sciences Research Council, Sweetwaters, South Africa; ^4^Department of Global Health, University of Washington, Seattle, WA, United States; ^5^MRC/Wits Developmental Pathways for Health Research Unit (DPHRU), University of the Witwatersrand, Johannesburg, South Africa; ^6^Institute for Global Health, University College London, London, United Kingdom; ^7^Department of Medicine, Division of Infectious Diseases, University of Washington, Seattle, WA, United States; ^8^Division of Infectious Diseases, Massachusetts General Hospital, Boston, MA, United States; ^9^Harvard Medical School, Boston, MA, United States

**Keywords:** HIV, HIV self-testing, secondary distribution, community, Sub-Saharan Africa, South Africa

## Abstract

**Background:**

To reach 95% of persons living with HIV (PLWH) knowing their HIV status, alternative testing approaches such as HIV self-testing (HIVST) and secondary HIVST kit distribution are needed. We investigated if secondary HIVST kit distribution from male and female PLWH in South Africa would successfully lead to their contacts testing for HIV and linking to care if positive.

**Methods:**

Male and female PLWH participating in an HIV treatment trial between July and November 2018 in KwaZulu-Natal, South Africa were offered participation as “HIVST kit distributors” in a pilot of secondary distribution of HIVST kits to give to sexual partners and social networks. Univariate descriptive statistics were used to describe the characteristics of volunteer distributors, proportion of HIVST recipients who reported their results, and linkage to care among those who tested positive using HIVST were assessed.

**Results:**

Sixty-three participant kit distributors accepted kits to disperse to contacts, of whom 52% were female, median age was 34 years (IQR 26-42.5), 84% reported 1 sexual partner and 76% did not know their partner's HIV status. HIVST kit distributors took 218 kits, with 13/218 (6%) of kits reported to be intended to be given to a sexual partner. A total of 143 HIVST recipients reported their HIVST results; 92% reported their results were negative, 11 recipients reported positive results and 1 HIVST-positive recipient was linked to HIV care.

**Conclusion:**

Secondary distribution of HIVST to social networks and sexual partners from South African PLWH is feasible, with two thirds of contacts reporting use of the HIVST kits. Additional support is necessary to facilitate linkage to care.

## Introduction

South Africa is home to the highest number of PLWH^1^ and KwaZulu–Natal (KZN) province has the highest HIV prevalence nationally (1)^2^. South Africa's HIV testing and care cascade stands at 92-70-64^3^, highlighting the great progress made in terms of people knowing their HIV status, but more effort is still needed to reach the United Nations Programme on HIV/AIDS (UNAIDS) 95-95-95 targets by 2025^4^. Individuals younger than 35 and men account for most of the people untested for HIV in KZN (2). Inconvenient clinic operating times, stigma, and preference for using traditional medicine are some of the barriers which prevent men from accessing facility-based HIV testing services (HTS) (3, 4). Alternative strategies to facility-based HTS are needed to reach these hard-to-reach populations.

HIV self-testing (HIVST), using a simple oral-fluid or blood-based self-test at a time and place convenient to the person testing, is a promising alternative strategy that addresses many barriers associated with facility-based HTS and has the potential to increase testing among persons who do not usually go to clinics (5). Different HIVST kit distribution strategies such as distributing through peer adolescent networks (6) and distributing to female sex workers (7) can be used to reach vulnerable populations who may experience barriers to accessing facility-based testing.

Secondary distribution of HIVST, where an HIVST kit is given to an individual for distribution to a third party, has been shown to reach male partners of women attending health facilities and can facilitate couples-testing (8, 9). Secondary HIVST kit distribution by peers in fishing communities was successful at finding untested men in one study in Uganda (10). In Sub-Saharan Africa, secondary kit distribution studies have primarily engaged women to distribute HIVST to partners and contacts (8, 9, 11). Little is known about secondary HIVST kit distribution using index distributors of both male and female PLWH as distributors in South Africa. We aimed to assess the willingness of both male and female PLWH to distribute HIVST kits to social networks including sexual partners, and to determine kit usage, test positivity, and linkage outcomes from this distribution approach.

## Methods

### Study Design

This was an observational cohort study nested within the Delivery Optimization of Antiretroviral Therapy (DO ART) trial of differentiated ART delivery in KZN, South Africa (12). Between July and November 2018, all DO ART participants attending follow-up visits in the peri-urban district of uMgungundlovu and the rural district of uMkhanyakude, were offered a choice of either oral swab (OraSure, OraQuick) or blood-based (i-Test, Atomo) HIVST kits to give to their adult (age 18 years and older) contacts. HIVST kit distributors (adult male or female PLWH) were informed that contacts could be a partner, relative, friend or neighbour. We did not restrict the gender of HIVST kits recipients. HIVST kit distributors were allowed to take up to 4 kits. When receiving the HIVST kits, HIVST kit distributors were given a brief instruction on how to use and demonstrate use of the kits to kit recipients. They were asked who they intended to give the kits to, and they were also given links to instructional videos which they could show or watch on their phones. The HIVST kits had instructions in the local language to call the study phone number when the kit recipient had completed the test and was ready to give a result. Following a similar approach used in the HIVST sub-study, an airtime voucher valued at 2 USD was provided to kit recipients at the time they reported results, as an incentive to provide HIVST results (5). The HIVST kit distributors did not do any communication of results to study staff.

### Study Procedures

The HIVST kit distributors completed a questionnaire while being issued HIVST kits. The questionnaire included topics such as their marital status, number of sexual partners, highest educational level completed, occupation, partner HIV status, intended kit recipients and number of kits taken. Kit recipients who completed the test and contacted the study team were taken through a telephonic questionnaire about demographic and clinical details. The kit recipients who called back with results were given the appropriate counselling dependent on the result. Those who tested negative received counselling on HIV prevention and those tested positive were counselled to go to the nearest clinic for confirmatory testing with two rapid HIV tests according to the national algorithm, and linkage to ART, or come to the DO ART study for confirmatory testing and enrolment for linkage to ART. A second phone outreach was conducted to kit recipients who reported a positive HIVST result to assess linkage to care. Study staff confirmed with clinic ART registries all participants who self-reported linkage to care. Study staff also conducted an ART registry search of Department of Health clinics to identify any late-linking persons after the reporting period concluded.

### Statistical Analysis

Univariate descriptive analyses were conducted and presented in percentages. Key demographic and clinical characteristics of the kit distributors were described using percentages. The main outcome of interest was the “return rate,” or proportion of distributed kits whose results were reported to the study. We then characterized the remainder of the “HIVST cascade.” We determined the proportion of returned HIVST results that were positive, and the proportion of persons with reactive HIVST results who subsequently linked to care for confirmatory testing and initiation of ART.

## Ethics

The HIVST kit distributors provided written informed consent to participate in the DO ART study and a separate consent to receive and distribute HIVST kits. Informed consent was obtained from secondary kit recipients over the phone once they called to give results after using the HIVST kit. Consent to use the test was implied if kit recipients contacted the study to report their results. This study received ethical approval from the Human Sciences Research Council, the University of KwaZulu-Natal and the University of Washington's Ethical Committees.

## Results

A total of 63 HIVST kit distributors took 218 HIVST kits to distribute to secondary contacts with a median of 4 kits (3 blood and 1 oral based) taken per participant. The HIVST kit distributors dispersing kits were 48% (*n* = 30) male and 52% (*n* = 33) female. The HIVST kit distributors median age was 34 years (26–42.5). Fifty-five percent (*n* = 35) indicated they were in a relationship and 84% (*n* = 52) indicated having 1 sexual partner (Table 1). Seventy five percent (*n* = 47) of those who took kits for distribution were unemployed; 89% (*n* = 54) reported an educational level at secondary or above. Seventy six percent (*n* = 48) indicated not knowing their partner's HIV status and 46 (73%) kits were reported to be intended to be given to a friend; 13 (6%) kits were intended to given to a sexual partner.

**Table 1:** Kit distributor characteristics.

**Kit distributors (*****n*** **= 63)**			
Gender	Male	30	(48%)
	Female	33	(52%)
Age, median (IQR)		34	(26–42.5)
	18–29	24	(38%)
	30–49	37	(59%)
	50+	2	(3%)
Marital status	Married	3	(5%)
	In relationship	35	(55%)
	Single	25	(40%)
Number of current sex partners	0	2/62	(3%)
	1	52/62	(84%)
	≥2	8/62	(13%)
Education	Primary	7/61	(11%)
	Secondary and above	54/62	(89%)
Occupation	Employed	16	(25%)
	Unemployed	47	(75%)
Partner HIV status	Positive	5	(8%)
	Negative	10	(16%)
	Unknown	48	(76%)
No. of kits taken, median (IQR)	Total	4	(2.5–4)
	OraQuick (Oral-based)	3	(1–4)
	Atomo (Blood-based)	0	(0–1)
Planned kit recipients (per kit, *N* = 218)	Sexual Partner Family member Friend Neighbour *Child Unknown	13 62 98 39 3 3	(6%) (29%) (46%) (39%) (3%) (3%)

* *Kits were intended to be distributed to adults; a proportion of the HIVST kit distributors indicated wanting to give the kits to their adolescent/adult children*.

**Figure 1:**
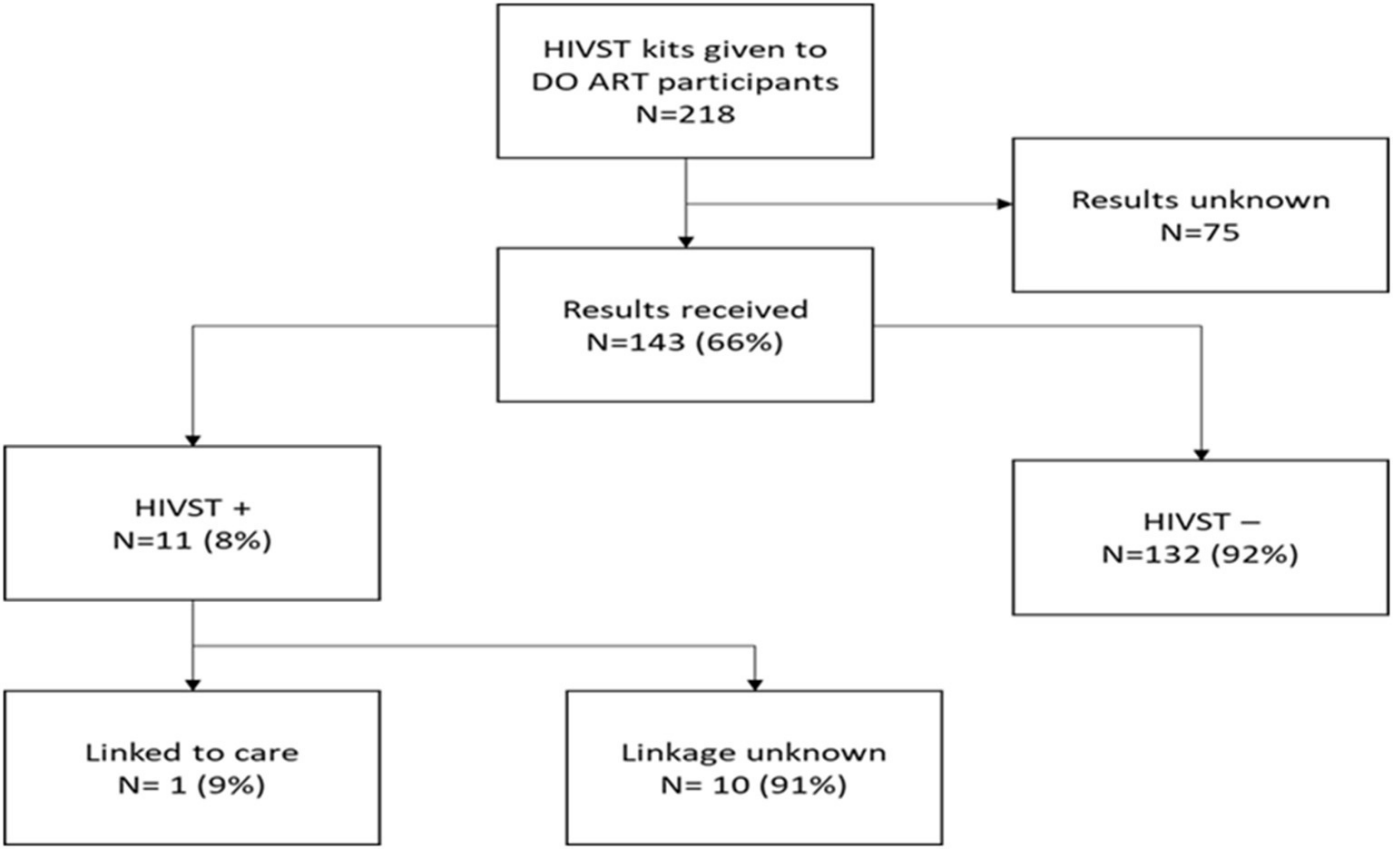
Secondary HIVST kit distribution cascade.

A total of 147/218 (67%) intended kit recipients contacted the study after using the kits. Recipients were [65 (44%)] males and [82 (56%)] females. Eighty-five recipients (58%) reported having previously tested for HIV prior to using the HIVST kit, and 143/147 (97%) reported their HIVST results. Four (3%) participants had missing data and therefore were recorded as not reporting their results (Table 2). Most of the HIVST results reported were negative at 92% (*n* = 132) (Figure 1). From the 11 (8%) recipients who reported positive HIVST results, only 1 (9%) reported being linked to care.

**Table 2:** Kit recipient characteristics.

Reported demographics		147/218	(67%)
Kit recipient gender	Male	65/147	(44%)
	Female	82/147	(56%)
Ever tested for HIV before	Yes	85/147	(58%)
	No	62/147	(42%)
Reported HIVST results	Yes	143/147	(97%)
	No	4/147	(3%)
HIVST result	Positive	11/143	(8%)
	Negative	132/143	(92%)
	Indeterminate	0/143	(0%)
Reported linkage	Yes	1/11	(9%)
	No	10/11	(91%)
Linked to care	Yes	1/1	(100%)
	No	0/1	(0%)

*Statistics are n (%) or median (interquartile range).

## Discussion

In this evaluation of secondary distribution of HIVST kits by male and female PLWH, we found that secondary kit distribution achieved a “return rate” of 143 of 218 (66%) kits used with results reported. The outcome of 75 (34%) kits remained unknown. From results reported, 62/147 (42%) indicated being first time testers, highlighting the potential secondary HIVT kit distribution has to find first-time testers. Even though 11 (8%) kit recipients reported to have tested positive, only 1 (9%) linked to care, leaving 10 (91%) with unknown linkage. This highlights the fact that HIVST can get people tested, but more support is needed to fully link them to care.

In finding first-time testers, our results are similar to findings by Choko et al. (10) who reported that secondary kit distribution using fishermen peers reached 23/89 (26%) men who had not previously tested for HIV in Uganda. Finding first-time testers will be an important step to overcome if the UNAIDS first “95” testing cascade will be achieved in KZN by 2025. Secondary kit distribution could be used an alternative strategy to find those not previously tested.

One challenge in evaluating secondary kit distribution is the reliance on testers to self-report their results, thus the number of HIVST results we received is an underestimate of the number of tests actually used. In their study of secondary kit distribution, Choko et al. (10) found that 87/94 (86%) participants recruited through fishermen peers in Uganda, reported their HIVST results. This reported percentage is much higher than the 66% reporting we found, but a major difference between the two studies is that the peers who distributed the kits in Uganda had to bring back the kits irrespective if they had been used. In contrast Hensen et al. (13) reported that 460/825 (56%) kit recipients confirmed to have used the kits after secondary distribution by community HIV care providers (CHIPs) in 4 communities within Zambia. In this study, kit recipients also had to return the kits to the CHIPs whether used or not. Across all studies, more kits were used and had results reported compared to unused/unknown kits, regardless of the reporting approach. We recommend that HIVST through secondary kit distribution is sufficiently high yield that it should be scaled up, even with the understanding that not all kits may be used or lead to immediate engagement in care.

From the HIVST kit distributors who dispersed kits, 48 (76%) indicated not knowing their partners' HIV status, yet only 13 (6%) kits were intended to distributed to a sexual partner. These results contrast with findings from Masters et al., who reported that secondary kit distribution by women seeking antenatal and postpartum care in Kenya promoted partner testing (8). Potential reasons for partners not wanting to distribute kits to partners could be that positive results can invite stigma, abandonment, discrimination, violence, and emotional distress (14). Secondary distribution of HIVST kits by PLWH to their sexual partners may result in disclosure of the distributor's HIV status. Additional elements could be incorporated into a program of HIVST distribution to social contacts in order to minimize social harms associated with status disclosure and promote partner kit distribution. Thirumurthy et al. (9) described an approach where secondary kit distributors were taught to use discretion when introducing self-tests to sexual partners by assessing likely reactions of their partner(s) as well as the risk of intimate partner violence. Scale-up of secondary kit distribution in the region should explore using these strategies.

An overwhelming majority of those who reported testing positive did not link to care. Future kit distribution programs should strengthen follow up and linkage to encourage those who test positive to easily link to care. Future research should explore reasons why people who reported to test positive through secondary HIVST kit distribution do not link to care.

This study had several limitations, including that we had no way to contact the kit recipients. We could not confirm if the HIVST kit distributors' intended kit recipients were the ones who received the kits. Also, even though two thirds (66%) of the participants responded with their results, this percentage is lower than the 80% responder percentage reported by a concurrent study providing primary distribution of HIVST kits to recipients in the same areas (5). Some kits may have been discarded before they were distributed, or recipients may not have used their kits. We were unable to ascertain results of kits that were used, or characteristics of the person who used the kit, unless the recipient elected to contact the study. Finally, self-reported HIVST results and linkage data are subject to some bias. If kit recipients already knew their status but had not disclosed to the distributor, this could have led to them accepting a test but not using it or not reporting their results.

The strength of the study is that it was done in an operationally friendly way using an existing structure of a community-based study, so it didn't have to create an entirely new infrastructure. The limited infrastructure requirements of this project suggest that results would be similar if translated into a public health setting, for example, implementing a secondary distribution programme in existing HIV treatment sites.

## Conclusions

We found that secondary kit distribution using both male and female PLWH as HIVST kit distributors can engage social contacts into HIV testing. However, low subsequent linkage to care among persons testing positive indicates further support with linkage is needed. This strategy can extend testing from facility-based into the community and reach first-time testers, and further efforts to improve linkage should be explored for persons accessing HIVST.

## Data Availability Statement

The raw data supporting the conclusions of this article will be made available by the authors, without undue reservation.

## Ethics Statement

This study received ethical approval from the Human Sciences Research Council, the University of KwaZulu-Natal and the University of Washington's Ethical Committees. The patients/participants provided their written informed consent to participate in this study.

## Author Contributions

AS, CC, and RB designed the study. NS, OK, KS, MK, and AV conducted the project and collected the data. NS, AS, and TS analyzed the data. NS and AS drafted the brief report. All authors contributed to the revisions and content of the final manuscript.

## Funding

This work was generously funded by the Bill and Melinda Gates Foundation (BMGF #OPP1134599).

## Conflict of Interest

The authors declare that the research was conducted in the absence of any commercial or financial relationships that could be construed as a potential conflict of interest.

## Publisher's Note

All claims expressed in this article are solely those of the authors and do not necessarily represent those of their affiliated organizations, or those of the publisher, the editors and the reviewers. Any product that may be evaluated in this article, or claim that may be made by its manufacturer, is not guaranteed or endorsed by the publisher.

## Footnotes

^1^ https://www.unaids.org/en/regionscountries/countries/southafrica

^2^ https://www.unaids.org/en/resources/presscentre/featurestories/2019/june/20190628_south-africa-modelling

^3^ https://www.unaids.org/sites/default/files/media_asset/2020_aids-data-book_en.pdf

^4^ https://aidstargets2025.unaids.org/#section-targets

## References

[1] Shisana O Rehle T Simbayi LC Zuma K Jooste S Zungu N. South African National HIV Prevalence, Incidence and Behaviour Survey, 2012. Cape Town: HSRC Press (2014). 10.2989/16085906.2016.1153491 27002359

[2] Huerga H Van Cutsem G Farhat JB Reid M Bouhenia M Maman D. Who needs to be targeted for HIV testing and treatment in KwaZulu-Natal? Results from a population-based survey. J Acquir Immune Defic Syndr. (2016) 73:411. 10.1097/QAI.0000000000001081 27243903 PMC5172512

[3] Beck D. Men and ARVs: How Does Being a Man Affect Access to Antiretroviral Therapy in South Africa? An Investigation Among Xhosa-Speaking Men in Khayelitsha. Cape Town: University of Cape Town (2004).

[4] Bassett IV Coleman SM Giddy J Bogart LM Chaisson CE Ross D. Barriers to care and 1-year mortality among newly diagnosed HIV-infected people in Durban, South Africa. J Acquir Immune Defic Syndr. (2017) 74:432–8. 10.1097/QAI.0000000000001277 28060226 PMC5321110

[5] Shapiro AE van Heerden A Krows M Sausi K Sithole N Schaafsma TT. An implementation study of oral and blood-based HIV self-testing and linkage to care among men in rural and peri-urban KwaZulu-Natal, South Africa. J Int AIDS Soc. (2020) 23:e25514. 10.1002/jia2.25514 32589337 PMC7319114

[6] Shahmanesh M Mthiyane TN Herbsst C Neuman M Adeagbo O Mee P. Effect of peer-distributed HIV self-test kits on demand for biomedical HIV prevention in rural KwaZulu-Natal, South Africa: a three-armed cluster-randomised trial comparing social networks versus direct delivery. BMJ Glob Health. (2021) 6:e004574. 10.1136/bmjgh-2020-004574 34315730 PMC8317107

[7] Chanda MM Ortblad KF Mwale M Chongo S Kanchele C Kamungoma N. HIV self-testing among female sex workers in Zambia: a cluster randomized controlled trial. PLoS Med. (2017) 14:e1002442. 10.1371/journal.pmed.1002442 29161260 PMC5697803

[8] Masters SH Agot K Obonyo B Napierala Mavedzenge S Maman S Thirumurthy H. Promoting partner testing and couples testing through secondary distribution of HIV self-tests: a randomized clinical trial. PLoS Med. (2016) 13:e1002166. 10.1371/journal.pmed.1002166 27824882 PMC5100966

[9] Thirumurthy H Masters SH Mavedzenge SN Maman S Omanga E Agot K. Promoting male partner HIV testing and safer sexual decision making through secondary distribution of self-tests by HIV-negative female sex workers and women receiving antenatal and post-partum care in Kenya: a cohort study. Lancet HIV. (2016) 3:e266–e274. 10.1016/S2352-3018(16)00041-2 27240789 PMC5488644

[10] Choko AT Nanfuka M Birungi J Taasi G Kisembo P Helleringer S. pilot trial of the peer-based distribution of HIV self-test kits among fishermen in Bulisa, Uganda. PLoS ONE. (2018) 13:e0208191–e0208191. 10.1371/journal.pone.0208191 30496260 PMC6264512

[11] Bulterys MA Mujugira A Nakyanzi A Nampala M Taasi G Celum C. Costs of providing HIV self-test kits to pregnant women living with HIV for secondary distribution to male partners in Uganda. Diagnostics. (2020) 10:318. 10.3390/diagnostics10050318 32438594 PMC7277977

[12] Barnabas RV Szpiro AA van Rooyen H Asiimwe S Pillay D Ware NC. Community-based antiretroviral therapy versus standard clinic-based services for HIV in South Africa and Uganda (DO ART): a randomised trial. Lancet Global Health. (2020) 8:e1305–15. 10.1016/S2214-109X(20)30313-2 32971053 PMC7527697

[13] Hensen B Schaap AJ Mulubwa C Floyd S Shanaube K Phiri MM. Who accepts and who uses community-based secondary distribution HIV self-testing (HIVST) kits? Findings from the intervention arm of a cluster-randomized trial of HIVST distribution nested in four HPTN 071 (PopART) communities in Zambia. J Acquir Immune Defic Syndr. (2020) 84:355–64. 10.1097/QAI.0000000000002344 32195749 PMC7340225

[14] Obermeyer CM Baijal P Pegurri E. Facilitating HIV disclosure across diverse settings: a review. Am J Public Health. (2011) 101:1011–23. 10.2105/AJPH.2010.300102 21493947 PMC3093267

